# Student Outcomes From a Large-Enrollment Introductory Course-Based Undergraduate Research Experience on Soil Microbiomes

**DOI:** 10.3389/fmicb.2021.589487

**Published:** 2021-07-27

**Authors:** Stanley M. Lo, Bryan D. Le

**Affiliations:** ^1^Section of Cell and Developmental Biology, University of California, San Diego, La Jolla, CA, United States; ^2^Division of Biological Sciences, University of California, San Diego, La Jolla, CA, United States; ^3^Program in Mathematics and Science Education, University of California, San Diego, La Jolla, CA, United States

**Keywords:** course-based undergraduate research experience, introductory biology, laboratory education, large enrollment, soil microbiome

## Abstract

In recent years, national reports have called for undergraduate laboratory education that engages students in authentic research experiences. As a result, a number of course-based undergraduate research experiences (CUREs) have been developed in biological sciences and some specifically in microbiology. Students benefit from CUREs much like in traditional mentored research experiences, where students carry out independent projects in faculty laboratories. These benefits include increased self-efficacy in research skills, enhanced identification as scientists, and higher graduation rates in science, technology, engineering, and mathematics majors. Because mentored research experiences are not readily available to every student, CUREs represent a potential mechanism to democratize the research experience by providing such opportunities to all students. However, many of existing CUREs described in the literature are designed for advanced undergraduates or are limited to a small number of students. Here, we report student outcomes from a large-enrollment introductory CURE on soil microbiomes that engages students in a real-world context with microbiology. In pre- and post-course surveys, students reported significant gains in self-efficacy on a number of research skills. These results are triangulated with post-course survey data on project ownership, sense of community, and CURE design elements such as collaboration, iteration, discovery, and relevance.

## Introduction

Research and laboratory experiences are important aspects of undergraduate education in biological sciences. In the past few decades, many national reports have called for the incorporation of research experiences into undergraduate education. Following broad calls in Science for All Americans (American Association for the Advancement of Science [AAAS], 1989) and Reinventing Undergraduate Education (Boyer Commission, 1998), more specific recommendations in biological sciences began to emerge: engaging students in the excitement of discoveries (National Research Council [NRC], 2003) and incorporating research experiences into laboratory courses in the first 2 years of the undergraduate curriculum (President’s Council of Advisors on Science, and Technology [PCAST], 2012). Recently, calls for transforming undergraduate education more broadly in science, technology, engineering, and mathematics (STEM) have increasingly focused on students across diverse educational contexts (National Academy of Sciences [NAS] et al., 2011; National Academies of Sciences, Engineering, and Medicine [NASEM], 2016).

Research experiences lead to improved outcomes for undergraduate students in many domains, such as disciplinary knowledge and competencies, professional and personal skills, identification as scientists, and persistence and time to degree in STEM (Hunter et al., 2006; Kinkel and Henke, 2006; Desai et al., 2008; Edwards et al., 2011; President’s Council of Advisors on Science, and Technology [PCAST], 2012; Horowitz and Christopher, 2013; Palmer et al., 2015). These outcomes are disproportionately beneficial for students from minoritized demographics, such as women and underrepresented minorities (Summers and Hrabowski, 2006; Summers, 2011; President’s Council of Advisors on Science, and Technology [PCAST], 2012). The Association of American Colleges and Universities considers undergraduate research experiences a high-impact educational practice that has been “widely tested and shown to be beneficial of college students from many backgrounds” (Kuh, 2008).

Mentored research experiences are available to a limited number of students. Especially at large public research universities, it is logistically infeasible for every undergraduate student to engage in mentored research experiences in faculty laboratories, simply given the student-to-faculty ratio. For example, at our institution, there are over 5,000 undergraduates majoring in biological sciences, with only about 100 faculty in the Division of Biological Sciences. Course-based undergraduate research experiences (CUREs) can be designed as part of the standard undergraduate laboratory curriculum, thus serving as a mechanism to make research experiences accessible to all students (Auchincloss et al., 2014). CUREs represent a democratization of the research experience by providing such opportunities to a much larger number of students, including students who belong to minoritized groups that have been historically underrepresented in science (Bangera and Brownell, 2014).

Course-based undergraduate research experiences engage students in scientific inquiry (Buck et al., 2008; Weaver et al., 2008) and are defined by a number of design elements: utilizing scientific practices, engaging with the collaborative and iterative nature of research, and making novel discoveries with broader relevance (Brownell and Kloser, 2015; Corwin et al., 2015a). Students benefit from CUREs much like in mentored research experiences, including increased self-efficacy in research skills, enhanced identification as scientists, and higher graduation rates in STEM majors (Lopatto et al., 2008; Shapiro et al., 2015; Rodenbusch et al., 2016). Many CUREs in biological sciences have been developed in the existing literature, such as annotating genome sequences, examining abiotic and biotic factors in ecology, investigating drug resistance in proteins (Chen et al., 2005; Taylor et al., 2010; Kloser et al., 2011, 2013). Examples in microbiology include discovering antibiotics, identifying bacteriophages, examining biofilms, and synthesizing biofuels (Hanauer et al., 2006; Davis et al., 2017; Pedwell et al., 2018; Light et al., 2019). However, many of these CUREs are for advanced undergraduates (Caspers and Roberts-Kirchhoff, 2003; Taylor et al., 2010; Butler et al., 2014; Murthy et al., 2014), and some are limited to a small number of students (Kloser et al., 2011, 2013; Thompson et al., 2016; Bhatt and Challa, 2018).

Previously, we reported the design and implementation of a large-enrollment introductory CURE on soil microbiomes that engages students in a real-world context with microbiology (Lo and Mel, 2017; Lo and Mordacq, 2020). Students work in teams to collect soil samples from native and invasive plant species at a biodiversity hotspot (Myers et al., 2000), compare soil properties such as moisture and pH, characterize microbial genetic biodiversity by 16S rRNA gene sequencing, and perform colorimetric assays to determine carbon source utilization of different soil microbiomes. Student teams also develop research proposals that they present at a poster conference to compete for mock grant funding. In this paper, we describe student outcomes from this CURE, including self-efficacy on research skills, project ownership, and sense of community.

## Materials and Methods

### Course Context

This study was conducted in the United States at a 4-year, public not-for-profit, and large doctoral university, described by The Carnegie Classification of Institutions of Higher Education (McCormick and Zhao, 2005) in the category of “very high research activity” and with a full-time, more selective, and higher transfer-in undergraduate profile. Human subject research was approved by the Institutional Review Board at the University of California San Diego. The CURE in this paper is part of the Introductory Biology Laboratory course at the study institution, which is a stand-alone course without prerequisites and not associated with lecture-based courses. Laboratory sections in the course meet once a week, and all learning activities are connected with the soil microbiome project in the CURE.

We define authentic research experiences in our CURE using the situated learning theory, which posits that learning takes place in the same context in which it is applied (Lave and Wenger, 1991) and as part of a community of practice (Wenger, 1999). Situated learning occurs through a process called legitimate peripheral participation (Lave and Wenger, 1991), meaning that students engage in the same tasks that scientists would do in a real research setting (“legitimate”), even though students may be performing at a less complex or sophisticated level (“peripheral”). Specifically, students collaborate in research projects that can result in novel conclusions with broader relevance, and they engage in the iterative nature of scientific inquiry (Table 1).

**TABLE 1:** CURE design elements.

Design element	Course structure
Scientific practices	Students collect and analyze data to draw conclusions
Collaboration	Teams of students collaborate and share research data
Iteration	Previous results are incorporated into assignments
Discovery	Novel soil microbiome data are collected by students
Relevance	Research question is of interests to professional scientists

*Specific course structure and activities were developed in alignment with the CURE design elements described in the existing literature: scientific practices, collaboration, iteration, discovery, and relevance.*

### Study Samples

Pre-course surveys were given in the first 2 weeks of the quarter. Post-course surveys were administered in the last 2 weeks of the quarter prior to final examinations. In our institutional context, this was the timeframe in which the student course evaluations were also administered on campus. In the past, we found that asking students to complete surveys after final examinations resulted in very low response rates. Therefore, we opted to administer the surveys for our study at the same time as the student course evaluations.

Survey data were collected over two academic quarters. In earlier implementations of the CURE, we observed many incomplete survey responses, and students expressed dissatisfaction with the number of surveys in the course, suggesting respondent fatigue (Ben-Nun, 2008). Therefore, we administered different subsets of surveys across the academic year (Table 2). While this approach resulted in a smaller data set, which reduces statistical power, we reasoned that the rotation of surveys could potentially yield a higher response rate and more meaningful responses. Historically, the overall grade distributions of the course have remained consistent across academic quarters over the years, suggesting minimal variations in the student populations that enroll in the course in different academic quarters.

**TABLE 2:** Survey administration response rates.

Survey instrument	Response rate
Classroom undergraduate research experience survey	165/248 (82.1%)
Project ownership survey	203/239 (85.4%)
Classroom community inventory	165/248 (82.1%)
Laboratory course assessment survey	203/239 (85.4%)

*Different subsets of surveys were administered across two academic quarters to minimize respondent fatigue.*

### Survey Instruments

Student outcomes were measured pre- and post-course by the classroom undergraduate research experience survey (Denofrio et al., 2007). We used a modified version of the classroom undergraduate research experience survey that changed the five-point scale on self-reported post-course learning gains (1 = no gain, 5 = very large gain) to a six-point scale on pre- and post-course self-efficacy (1 = no skill, 6 = very high skill) to capture the pre-course baseline. The six-point scale was intentionally chosen to eliminate the ambiguous mid-point option in the original five-point scale, which could be interpreted as neutral or undecided, two similar but distinct constructs (Komorita, 1963; Guy and Norvell, 1977; Armstrong, 1987). These modifications were previously determined to retain high internal consistency and reliability (Mordacq et al., 2017).

We also measured student outcomes using the project ownership survey (Hanauer and Dolan, 2014) and the classroom community inventory (Rovai et al., 2004). The laboratory course assessment survey (Corwin et al., 2015b) was also administered to capture student perspectives on whether specific CURE design elements such as collaboration, iteration, discovery, and relevance were present in the course. These three surveys were administered only at the end of the course (“post-course”), as they describe student experiences within the course, and the items would not make sense at the beginning of the course (“pre-course”). For these instruments, we used the various Likert or Likert-like scales in the original literature, some of which were on a five-point scale. This is based on recommendations to allow for neutral responses instead of forced directional choices for items especially related to emotions and affect (Komorita, 1963; Guy and Norvell, 1977; Armstrong, 1987).

### Statistical Analysis

Descriptive statistics were calculated for all survey responses. For the Classroom Undergraduate Research Experience survey, only matched pre-and-post pairs were included for analysis. Pre- and post-course responses were compared using the Wilcoxon signed-ranked test because of the non-parametric nature of the data (Wilcoxon, 1945), and the Holm-Bonferroni correction was used to correct for multiple comparisons (Holm, 1979; Shaffer, 1995). Effect sizes were calculated using Cohen’s d, which is defined as the difference between the pre- and post-course means normalized to the standard deviation from the pre-course data (Maher et al., 2013). For the items administered only post-course, analysis of variance with the Tukey’s honestly significant difference (HSD) test was used to determine if responses for items within each survey construct were statistically different. All statistical analyses were performed in JMP Pro Version 13.0–16.0 or Microsoft Excel.

## Results

Pre- and post-course results from the classroom undergraduate research experience survey showed that students reported self-efficacy gains in 22 out of 25 items (Table 3). In the category of research skills, significant gains (*p* < 0.05) in self-efficacy ranged from 0.16 to 0.85 in effect size across nine out of 10 items. Writing a research proposal and reading scientific literature showed the highest gains with effect sizes of 0.85 and 0.80, respectively, which are considered large (Maher et al., 2013). This large gain likely resulted from the course project in addition to the laboratory experiments on soil microbiomes, where student teams developed research proposals based on primary literature of interests to them. Performing computer calculations and maintaining a research notebook had effect sizes of 0.79 and 0.56, respectively, which are considered medium (Maher et al., 2013). Both were regular activities done in laboratory sections every week. Analyzing research data had a much smaller effect size of 0.24, despite also being part of the laboratory activities each week. This survey item may be less specific compared to performing computer calculations and maintaining a research notebook and thus did not resonate in students’ mind as something they had done regularly in the course. Critiquing work of other students and presenting results as papers had effect sizes of 0.42 and 0.24, which are considered small (Maher et al., 2013). These activities only occurred 3–4 times throughout the quarter and thus likely resulted in the smaller effect sizes. Surprisingly, no statistical difference was observed pre- and post-course for self-efficacy in presenting a poster, even though student teams presented their research proposals as posters in a conference format as their final examination. This was likely due to the timing of the survey administration, which was completed before the week of final examinations to encourage a higher response rate.

**TABLE 3:** Classroom undergraduate research experience survey.

Item	Pre	Post	*p*	ES
**Research skills**				
Write a research proposal	2.0 ± 0.9	2.7 ± 0.8	****	0.85
Read scientific literature	2.5 ± 0.8	3.1 ± 0.7	****	0.80
Perform computer calculations	1.6 ± 0.8	2.4 ± 0.9	****	0.79
Maintaining research notebook	3.0 ± 1.0	3.5 ± 0.8	****	0.56
Critique work of other students	2.8 ± 0.8	3.1 ± 0.7	****	0.42
Analyze research data	3.3 ± 0.7	3.5 ± 0.7	**	0.24
Present results in papers	3.1 ± 0.9	3.3 ± 0.8	**	0.24
Collect data	3.4 ± 0.7	3.5 ± 0.8	*	0.17
Present results orally	2.7 ± 0.9	2.9 ± 0.8	*	0.16
Present a poster	3.0 ± 1.0	3.0 ± 0.8	n.s.	0.05
**Doing a project where …**				
No one knows the outcome	2.0 ± 0.9	2.6 ± 0.9	****	0.64
Students have some input	2.5 ± 1.0	3.0 ± 0.8	****	0.61
Entirely designed by students	2.1 ± 0.9	2.7 ± 0.9	****	0.59
Students are responsible	3.8 ± 0.7	4.0 ± 0.7	**	0.28
Instructor knows the outcomes	3.0 ± 0.8	3.2 ± 0.8	**	0.28
Structured by the instructor	3.5 ± 0.9	3.7 ± 0.8	*	0.20
Students know the outcome	3.1 ± 0.8	3.3 ± 0.9	*	0.17
**General course skills**				
Work on problems	3.6 ± 0.7	4.0 ± 0.8	*	0.47
Listen to lectures	3.9 ± 0.7	4.2 ± 0.7	*	0.39
Work as a whole course	2.9 ± 0.8	3.2 ± 0.8	***	0.31
Take tests	4.1 ± 0.6	4.2 ± 0.7	*	0.27
Work in small groups	3.8 ± 0.6	3.9 ± 0.6	*	0.22
Discuss reading materials	3.4 ± 0.8	3.6 ± 0.7	*	0.20
Read textbook	3.9 ± 0.8	4.0 ± 0.8	n.s.	0.12
Work individually	3.5 ± 0.9	3.6 ± 1.0	n.s.	0.11

Items are grouped into three categories (research skills, experiences with different types of projects, and general course skills) and ordered by effect size (ES, calculated as Cohen’s d) from large to small within each category. Items are on a six-point Likert-like scale (1 = no skill, 6 = very high skill). Descriptive statistics (average ± standard deviation) are reported. Statistical differences are indicated by the following notation: *p < 0.05; **p < 0.01; ***p < 0.001; ****p < 0.0001; and n.s., not significant.

In the category of experience with different types of research projects, significant gains (*p* < 0.05) in self-efficacy ranged from 0.17 to 0.64 in effect size across all seven items. Students reported highest gains in doing a project where no one knows the outcome, where students have some input, and where entirely designed by students with effect sizes of 0.64, 0.61, and 0.59, respectively, which are considered moderate (Maher et al., 2013). In the course, we emphasized that the soil microbiome project was original research where the students would be the first to collect and analyze their data and that no other students had previously reported the same data. The CURE aspect of the course, along with the research proposals developed by student teams, likely resulted in these moderate effect size.

In the category of general course skills, six out of eight items showed significant gains (*p* < 0.05) with effect sizes ranging from 0.20 to 0.47, which are considered small (Maher et al., 2013). Many of these items were not directly related to the CURE aspects of the course, and students would have likely reported gains in working on problems, listening to lectures, and taking tests even if they were in a lecture course or a non-CURE laboratory course. Reading a textbook and working individually showed no statistical difference pre- and post-course. These were not activities emphasized in the course, as there was minimal reading other than the laboratory manual, and students always worked in teams of laboratory experiments and their research proposals.

In terms of project ownership (Table 4), students reported highest post-course ratings in the items “my research project was interesting” (average ± standard deviation = 4.2 ± 0.5 on a five-point Likert scale) and “my project gave me a sense of personal achievement” (3.9 ± 0.8). Students also reported lowest post-course ratings in the item “I had a personal reason for choosing the research project” (3.1 ± 1.9). The latter result was perhaps not surprising, as the soil microbiome project was relatively structured and not chosen by individual students given the large-enrollment nature of the course.

**TABLE 4:** Project ownership survey.

Item	Avg	SD	A	B	C	D
**Ownership**						
My research project was interesting	4.2	0.5	A			
My project gave me a sense of personal achievement	4.1	0.7	A			
I was responsible for the outcomes of my research	4.0	0.7	A	B		
In my project, I actively sought advice and assistance	4.0	0.8	A	B		
I faced challenges that I managed to overcome	3.9	0.6	A	B		
My findings were important to the scientific community	3.7	0.7		B	C	
My research will help to solve a problem in the world	3.7	0.7		B	C	
My research project was exciting	3.7	0.8			C	
The research question I worked on was important to me	3.5	0.8			C	
I had a personal reason for choosing the project	3.1	0.9				D
**Emotions**						
Delighted	3.7	0.8	A			
Happy	3.7	1.1	A			
Joyful	3.4	1.0	A			
Amazed	3.0	1.0		B		
Surprised	2.8	0.9		B		
Astonished	2.7	0.9		B		

*Items are grouped into two categories: project ownership (five-point Likert scale: 1 = strongly disagree, 5 = strongly agree) and emotions associated with project experience (five-point Likert-like scale: 1 = very slightly, 5 = very strongly). Average (Avg) and standard deviation (SD) are reported, and items are ordered by average from highest to lowest value within each category. Columns A-D: Items within each column are not statistically different, whereas items in different columns are statistically different, based on separate ANOVAs followed by Tukey’s HSD tests, one for ownership and one for emotions.*

For the results from the emotions items on a five-point Likert-like scale, students reported being delighted (3.7 ± 0.8), happy (3.7 ± 1.1), and joyful (3.4 ± 1.0) more so than being amazed (3.0 ± 1.0), surprised (2.8 ± 0.9), and astonished (2.7 ± 0.9). These results were similar to those from published sources (Hanauer and Dolan, 2014), with the exception of surprised and astonished, which were positive in the original study. In our CURE, the research project compared soil properties and microbiomes associated with native and invasive plant species. While the comparison was helpful in teaching basic hypothesis testing and statistics, there was simply no reason for students to envision *a priori* which soil sample would have a higher pH or more diverse microbiome. Correspondingly, it would seem reasonable that students were not surprised or astonished.

For classroom community, students reported 3.4 ± 0.7 and 3.1 ± 0.4 (on a five-point Likert scale) for the peer support and learning support dimensions, respectively (Table 5). Peer support includes items such as “I feel connected to others in this course” and “I feel that I can rely on others in this course.” Learning support includes items such as “I feel that I am given ample opportunities to learn in this course” and “I feel that my educational needs are not being met in this course” (reverse-coded item). These results are similar to those in the original literature, with ratings in peer support and learning support at 3.3 ± 0.5 and 2.9 ± 0.9, respectively (Rovai et al., 2004).

**TABLE 5:** Classroom community inventory.

Item	Avg	SD
Peer support	3.4	0.7
I trust others in this course	3.5	0.8
I feel that students in this course care about each other	3.4	0.8
I feel connected to others in this course	3.4	0.9
I feel confident that others in this course will support me	3.4	0.8
I feel that I can rely on others in this course	3.4	0.9
Learning support	3.1	0.4
I feel that I am given ample opportunities to learn in this course	3.7	0.8
I feel that I receive timely feedback in this course	3.6	0.8
* I feel that this course results in only modest learning	3.3	0.8
* I feel that my educational needs are not being met in this course	2.7	1.0
* I feel that this course does not promote a desire to learn	2.4	1.0

Items are grouped into two dimensions of peer support and learning support. Each dimension consists of five related items on a five-point Likert scale (1 = strongly disagree, 5 = strongly agree). Average (Avg) and standard deviation (SD) for each dimension and item are reported. Items with * are reverse coded, and ratings are reported after being converted to the positive scale.

The laboratory course assessment survey provides additional information on how students perceived the presence of three of the five CURE design elements: iteration, discovery, and collaboration (Table 6). Students reported average post-course ratings of 4.2 ± 0.8 and 4.1 ± 0.8 (on a six-point Likert-like scale) for the iteration and discovery dimensions, respectively. The iteration dimension includes items such as “share and compare data with other students,” and the discovery dimension includes items such as “develop new arguments based on data.” In the course, students completed 3–4 writing assignments, in which they constructed scientific arguments to draw conclusions based on data in the laboratory. These writing assignments asked students to use data collected and analyzed by all student teams in the course. In laboratory sections, student teams posted their data on Google Spreadsheet files to facilitate the sharing of data. Student teams were also asked compare their own data with those from other teams as they data were shared.

**TABLE 6:** Laboratory course assessment survey.

Item	Avg	SD
Iteration	4.2	0.8
Share and compare data with other students	4.7	0.8
Revise drafts of papers or presentations based on feedback	4.3	1.1
Revise or repeat analyses based on feedback	4.1	1.1
Collect and analyze additional data to address new questions	3.9	1.2
Change the methods of the investigation	3.8	1.2
Discovery	4.1	0.8
Formulate my own research questions or hypothesis	4.3	1.0
Develop new arguments based on data	4.2	1.0
Revise or repeat work to account for errors or fix problems	4.2	1.1
Explain how my work has resulted in new scientific knowledge	4.2	1.1
Conduct an investigation to find something previously unknown	4.1	1.0
Generate novel results that could be of interest to the community	3.6	1.2
Collaboration	3.5	0.6
Discuss elements of my investigation with classmates or instructors	3.7	0.6
Reflect on what I was learning with others	3.7	0.7
Share problems and seek input on how to address them	3.6	0.8
Contribute my ideas and suggestions during class discussions	3.5	0.8
Help other students collect or analyze data	3.4	0.9
Provide constructive criticism and challenge each other’s interpretations	3.1	1.0

*Items are grouped into three dimensions related to some of the CURE design elements: iteration, discovery, and collaboration. Items in the iteration and discovery dimensions are on a six-point Likert-like scale (1 = strongly disagree, 6 = strongly agree), and items in the collaboration dimension are on a four-time frequency scale (1 = never, 4 = weekly). Average (Avg) and standard deviation (SD) for each dimension and item are reported.*

For collaboration, which include items such as “discuss elements of my investigation with classmates or instructors” and “help other students collect or analyze data,” students reported a post-course rating of 3.5 ± 0.6 (on a four-point frequency scale: 1 = never, 2 = one or two times, 3 = monthly, and 4 = weekly). Laboratory sections met once for 3 h each week, and students always worked in teams to collect and analyze data. Student teams also developed their research proposals in a scaffolded fashion with dedicated work time in laboratory sections and milestones throughout the quarter. Therefore, it was likely that these items in the collaboration dimension have happened weekly or almost weekly.

## Discussion

In this paper, we report student outcomes from a CURE on soil microbiomes situated in a large-enrollment introductory biology laboratory course. Early research experiences are critical to student learning, as well as identity formation and persistence in STEM, and CUREs in introductory courses can play an important role in promoting student success (President’s Council of Advisors on Science, and Technology [PCAST], 2012). Compared to many other examples in the existing literature, this CURE is unique in two ways. The course is required for all biological sciences majors at the study institution and does not have any prerequisites, thus providing universal access to research experiences for all beginning undergraduate students before they are likely to encounter the negative weed-out environment common in introductory STEM courses (Mervis, 2011).

Learning activities in this soil microbiome course were intentionally developed based on the five CURE design elements (Table 1). The intended curriculum (designed by educators based on learning principles) can be substantially different from what students experience in the classroom (Bussey et al., 2013; Lloyd et al., 2017). Therefore, it is important to examine the student perspectives. In post-course surveys, students reported ratings in agreement with the presence of the CURE design elements. Three of the five design elements (collaboration, iteration, and discovery) were observed in the laboratory course assessment survey (Table 6). Certain items in the project ownership survey, including “my research will help to solve a problem in the world” and “my findings were important to the scientific community,” directly relate to relevance, and students reported agreement with the presence of this design element (Table 5). For scientific practices, students reported significant gains in self-efficacy on research skills, with effect sizes larger than gains in self-efficacy on general course skills (Table 4). In the category of experiences with different types of projects, students reported moderate effect sizes in the items related to doing a project where “no one knows the outcome,” “students have some input,” and “entirely designed by students” but only small effect sizes for projects where “instructor knows the outcomes,” “structured by the instructor,” and “students know the outcome” (Table 4), further suggesting the presence of the scientific practices CURE design element.

Student outcomes in this paper are primarily observed through pre- and post-course surveys on self-efficacy on research skills, and students reported significant gains in 22 out of 25 items from the classroom undergraduate research experience survey (Table 3). In parallel, within the course, students completed writing assignments and poster presentations that were graded as summative assessments to determine if they have achieved the course learning objectives, even though these artifacts were not included as part of this study. Furthermore, while self-efficacy is not the same as cognitive performance on assessment tasks, affective considerations are important for student persistence in STEM. In fact, students from minoritized backgrounds leave STEM majors at disproportionately higher rates compared to students from majority and dominant cultures, and this exclusion is not primarily related to academic performance (Seymour and Hewitt, 1997; Asai, 2020). Compared to two decades ago, a higher percentage of students today report negative teaching and learning experiences related to the affective domain as reasons for leaving STEM majors (Seymour and Hewitt, 1997; Seymour and Hunter, 2019). Self-efficacy is a key affective component in science identity (Carlone and Johnson, 2007; Hazari et al., 2013), and research experiences can help promote the development of science identity through increasing self-efficacy (Graham et al., 2013). Therefore, it is reasonable to expect that increased self-efficacy from CUREs such as the one described here will ultimately lead to higher persistence in STEM.

## Data Availability Statement

Data are available upon reasonable request and with permission of the Institutional Review Board.

## Ethics Statement

The studies involving human participants were reviewed and approved by University of California, San Diego, Human Research Protections Program. Written informed consent for participation was not required for this study in accordance with the national legislation and the institutional requirements.

## Author Contributions

SL designed the study, collected and analyzed data, and wrote the manuscript. BL analyzed data and generated tables. Both authors contributed to the article and approved the submitted version.

## Conflict of Interest

The authors declare that the research was conducted in the absence of any commercial or financial relationships that could be construed as a potential conflict of interest.

## Publisher’s Note

All claims expressed in this article are solely those of the authors and do not necessarily represent those of their affiliated organizations, or those of the publisher, the editors and the reviewers. Any product that may be evaluated in this article, or claim that may be made by its manufacturer, is not guaranteed or endorsed by the publisher.
